# Biooxidation of Ciguatoxins Leads to Species-Specific Toxin Profiles

**DOI:** 10.3390/toxins9070205

**Published:** 2017-06-29

**Authors:** Tsuyoshi Ikehara, Kyoko Kuniyoshi, Naomasa Oshiro, Takeshi Yasumoto

**Affiliations:** 1 Department of Food Science and Technology, National Fisheries University, 2-7-1 Nagata-honmachi, Shimonoseki, Yamaguchi 759-6595, Japan; 2 National Institute of Health Sciences, 1-18-1 Kamiyoga, Setagaya, Tokyo 158-8501, Japan; k-kuniyoshi@nihs.go.jp (K.K.); n-oshiro@nihs.go.jp (N.O.); 3 Japan Food Research Laboratories, 6-11-10 Nagayama, Tama, Tokyo 206-0025, Japan; yasumotot@jfrl.or.jp

**Keywords:** ciguatera, ciguatoxins, in vitro oxidation, fish liver S9, Cyp3A4

## Abstract

Ciguatoxins (CTXs) contaminate fish worldwide and cause the foodborne illness ciguatera. In the Pacific, these toxins are produced by the dinoflagellate *Gambierdiscus toxicus*, which accumulates in fish through the food chain and undergoes oxidative modification, giving rise to numerous analogs. In this study, we examined the oxidation of CTXs in vitro with liquid chromatography-tandem mass spectrometry (LC-MS/MS) analysis using reference toxins, and found that CTX4A, CTX4B, and CTX3C, which are produced by the alga, are oxidized to the analogs found in fish, namely CTX1B, 52-*epi*-54-deoxyCTX1B, 54-deoxyCTX1B, 2-hydroxyCTX3C, and 2,3-dihydroxyCTX3C. This oxidation was catalyzed by human CYP3A4, fish liver S9 fractions, and microsomal fractions prepared from representative ciguateric fishes (*Lutjanus bohar*, *L. monostigumus*, and *Oplegnathus punctatus*). In addition, fish liver S9 fractions prepared from non-ciguateric fishes (*L. gibbus* and *L. fulviflamma*) in Okinawa also converted CTX4A and CTX4B to CTX1B, 54-deoxyCTX1B, and 52-*epi*-54-deoxyCTX1B in vitro. This is the first study to demonstrate the enzymatic oxidation of these toxins, and provides insight into the mechanism underlying the development of species-specific toxin profiles and the fate of these toxins in humans and fish.

## 1. Introduction

Ciguatera fish poisoning (CFP) is a clinical syndrome that causes neurological symptoms in patients and affects more than 50,000 people per year [1,2,3,4,5]. This foodborne illness results from eating fish from warm water regions around the world that contain natural marine toxins that are collectively known as ciguatoxins (CTXs). CTXs are produced by the benthic dinoflagellate initially assigned *Gambierdiscus toxicus* with reassignment later to *G. polynesiensis*, enter the food chain via herbivorous fish and subsequently accumulate in the tissue of hundreds of species of fish [6,7,8].

**Figure 1:**
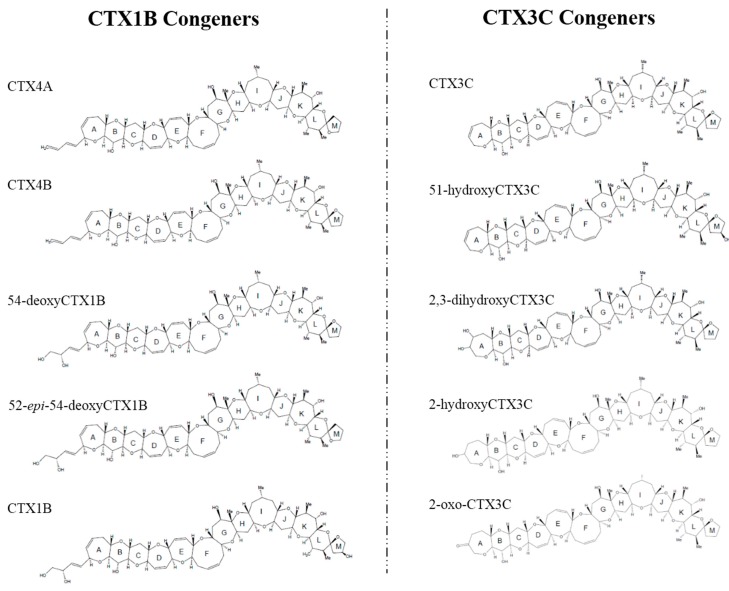
Structures of various ciguatoxins (CTXs).

Chemical studies performed on the dinoflagellate and fish from the Pacific have identified up to 23 analogs of CTXs, all of which contain contiguous ether rings aligned in a ladder shape [9,10]. However, the backbone skeletons of these toxins differ, allowing them to be classified into two distinctive types: CTX1B and CTX3C (Figure 1). It has been shown that the oxygenated congeners CTX1B and 54-deoxyCTX1B are more toxic than CTX4A and 4B in mice [7,11,12,13,14]. Furthermore, a distinction has been noted between the toxins that are produced by *G. toxicus* and those that are found in fish, strongly suggesting that the original toxins are metabolically-oxidized in fish, mostly at the terminal parts of the molecule; and a recent study showed that various fish from Okinawa and some other regions retained species-specific toxin profiles over a 20-year time period [15,16], implying that a genetic factor may be involved in shaping their toxin profiles. However, this hypothetical biooxidation of CTXs has remained untested due to the unavailability of pure toxins for use as substrates and for identifying the products.

Cytochrome p450 (CYP) enzymes constitute a superfamily of many different monooxygenases that play a pivotal role in drug metabolism [17]. These enzymes are present in all living organisms and are expressed in many tissues in vivo, but are most abundant in the mammalian liver. Thus, the liver S9 and microsomal fractions include drug metabolizing enzymes such as CYP enzymes, and are mainly used to test for the metabolism of alien substances, drug interactions, and the formation of covalent bonds between drugs [18,19,20].

In this study, we aimed to verify the bio-oxidation process of CTXs in vitro using CTX4A/4B and CTX3C as substrates, and recombinant human CYP3A4 (rhCYP3A4), fish liver S9 fractions, and microsomal fractions as oxidizing enzymes. The reaction products were identified by liquid chromatography-tandem mass spectrometry (LC-MS/MS) analysis using reference toxins, which unambiguously confirmed the production of CTX1B, 54-deoxyCTX4B, 52-*epi*-54-deoxyCTX1B, 2-hydroxyCTX3C, and 2,3-dihydroxyCTX3C. Our findings shed light on the mechanism that leads to the characteristic toxin profiles in fish species and also enrich our knowledge of the dynamic fate of these toxins.

## 2. Results

### 2.1. In Vitro Oxidation of CTXs by rhCYP3A4

**Figure 2:**
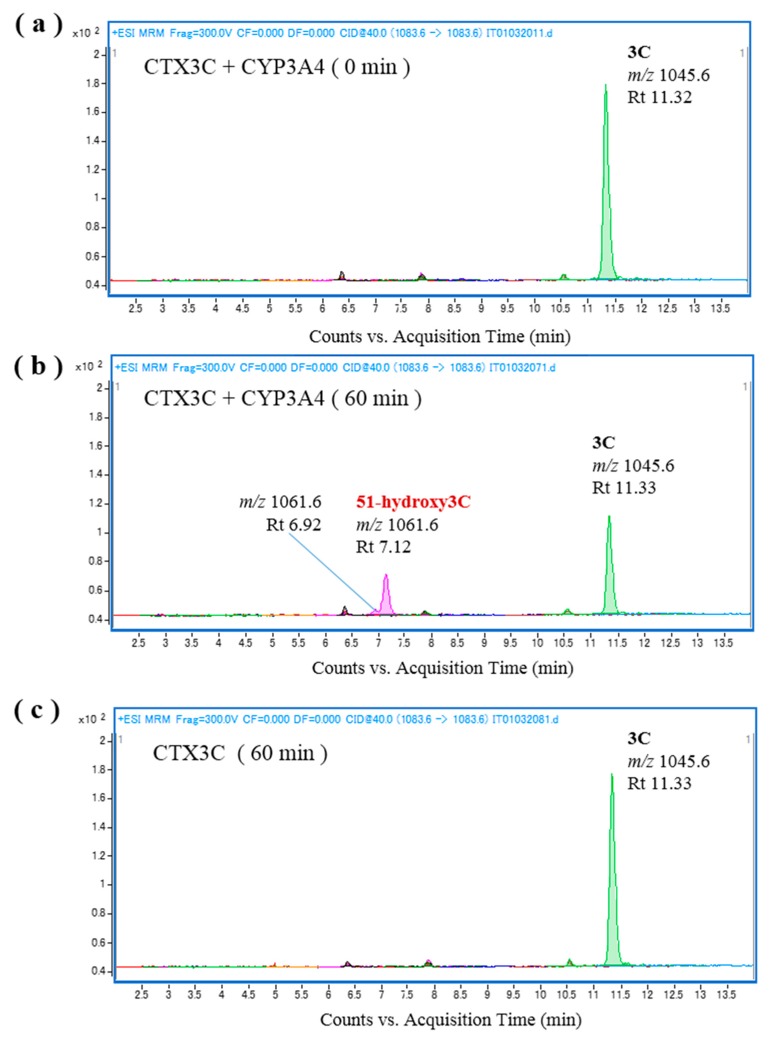
Chromatograms of the in vitro oxidation products of ciguatoxin-3C (CTX3C) following incubation with recombinant human CYP3A4 (rhCYP3A4) at 37 °C. Products are shown (**a**) at 0 min, (**b**) at 60 min, and (**c**) at 60 min without rhCYP3A4.

To verify the biooxidation of CTXs, we attempted to oxidize the substrates CTX4A, CTX4B, and CTX3C in vitro (Figure S1a,b), and identified the reaction products by LC-MS/MS analysis using reference toxins (Figure S1c). The abbreviations and the [M + Na]^+^ (*m*/*z*) value of each CTX congener is shown in Table 1. To establish the experimental conditions, we first attempted in vitro oxidation using rhCYP3A4 and human liver microsomes because CYP3A4 is one of many human CYP enzymes that plays a central role in drug metabolism, and biotransforms a wide range of endogenous and exogenous compounds including drugs, toxins, and pollutants. We found that incubation of CTX3C with rhCYP3A4 produced 51-hydroxyCTX3C (Figure 2); incubation of CTX4A/4B with rhCYP3A4 produced CTX1B, M-*seco*-CTX4A/4B, and 7-hydroxyCTX4A/4B (Figure S2); and incubation of CTX4A/4B with human liver microsomes including CYP3A4 produced CTX1B, 52-*epi*-54-deoxyCTX1B, 54-deoxyCTX1B, and M-*seco*-CTX4A/4B, none of which were detected when microsomes that had been inactivated by heat-treatment were used (Figure S3). These results indicate that rhCYP3A4 and human liver microsomes, including CYP3A4, can be successfully used for in vitro CTX oxidation.

### 2.2. In Vitro Oxidation of CTXs by Fish Liver S9 from Cigauteric and Non-Ciguateric Fishes

The CTX oxidation activity of fish liver extracts was examined using the experimental conditions that were established in the in vitro oxidation experiment (see above). We prepared fish liver S9 fractions from 18 fish specimens belonging to three representative ciguateric fish species (*L. bohar*, *L. monostigma*, and *O. punctatus*) and two non-ciguateric fish species (*L. gibbus* and *L. fulviflamma*) (Table S1). These fish were collected from the Ryukyu Islands in Okinawa Prefecture, where large carnivores are frequently toxic, but there have been no observed cases of CFP in *L. fulviflamma* and *L. gibbus* [21].

**Figure 3:**
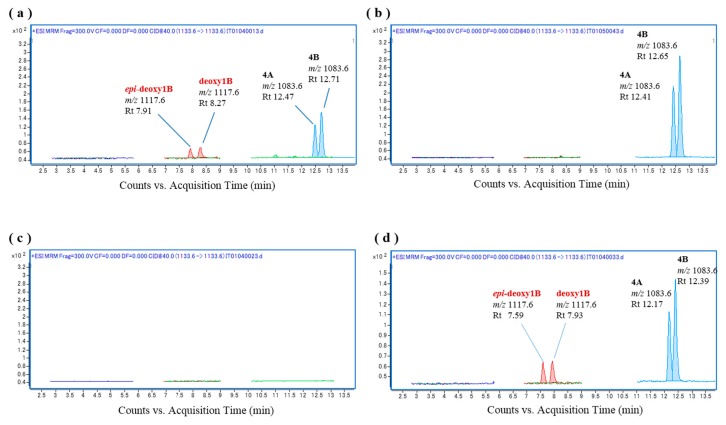
Chromatograms of the in vitro oxidation products of a ciguatoxin-4A/4B (CTX4A/4B) mixture following incubation with the liver S9 and microsomal fractions of *L. bohar*; (**a**) *L. bohar* liver S9 and CTX4A/4B, (**b**) CTX4A/4B, (**c**) *L. bohar* liver S9, and (**d**) *L. bohar* liver microsomal fraction and CTX4A/4B.

The fish liver S9 fraction was initially prepared from *L. bohar* liver homogenate because bio-oxidation activity in this fish was expected to be high due to snappers containing the oxidative analogs CTX1B, 52-*epi*-54-deoxyCTX1B, and 54-deoxyCTX1B, and *L. bohar* reportedly being the most common reef fish that is implicated in CFP poisoning in Okinawa [21]. As shown in Figure 3a, incubation of a mixture of the substrates CTX4A and CTX4B with *L. bohar* liver S9 as an oxidation catalyzing system at 37 °C for 60 min led to the production of 52-*epi*-54-deoxyCTX1B and 54-deoxyCTX1B, neither of which were contained in the substrates or the fish liver S9 fraction (Figure 3b,c). Oxidation of the CTXs was also detected when a further fractionated microsomal fraction was used (Figure 3d). These results indicate that 52-*epi*-54-deoxyCTX1B and 54-deoxyCTX1B were produced by the in vitro oxidation of CTX4A/4B by fish liver S9, suggesting that some of the CYP family enzymes that are abundant in the fish liver have oxidation activity.

**Figure 4:**
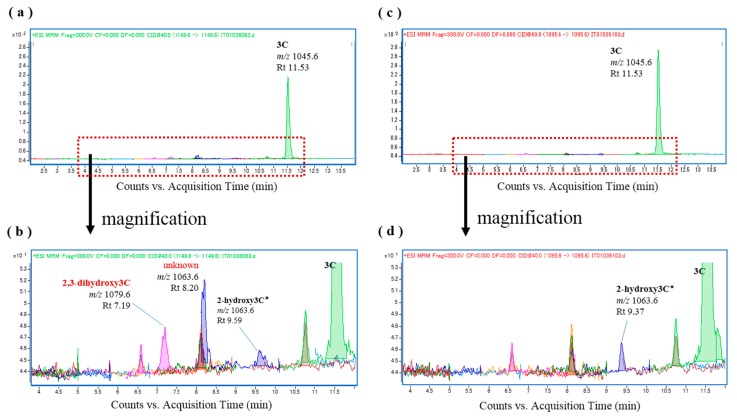
Chromatograms of the in vitro oxidation products of ciguatoxin-3C (CTX3C) following incubation with the liver S9 fraction of *L. bohar*; (**a**,**b**) *L. bohar* liver S9 and CTX3C; and (**c**,**d**) CTX3C.

To examine individual differences in CTX oxidation activity in *L. bohar*, liver S9 fractions were prepared from four additional specimens (see Table S1) and were used for in vitro CTX oxidation. In all samples but one, 52-*epi*-54-deoxyCTX1B and 54-deoxyCTX1B were again produced following incubation with the *L. bohar* S9 fractions (Figure S4a–d), and the fish liver S9 fractions were confirmed to be free of these toxins (Figure S5). Furthermore, when CTX3C was incubated with the *L. bohar* S9 fractions, 2,3-dihydroxyCTX3C was produced (Figure 4a,b) and it was confirmed that the CTX3C substrate was free of these analogs (Figure 4c,d). Tentative assignment in the absence of reference is shown with an asterisk.

**Figure 5:**
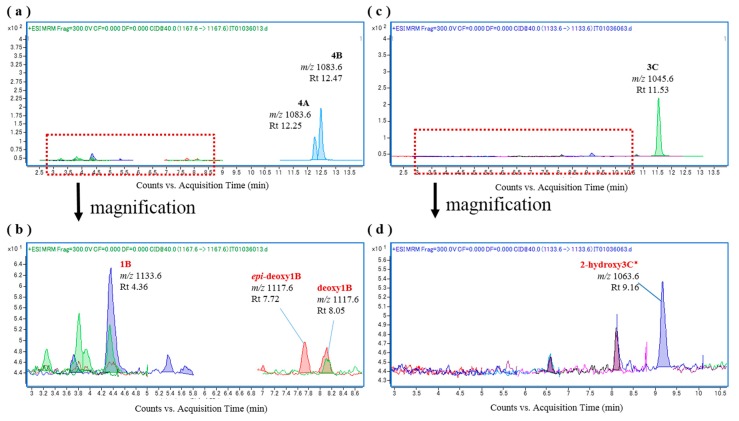
Chromatograms of the in vitro oxidation products of ciguatoxin-4A/4B (CTX4A/4B) and CTX3C following incubation with the liver S9 fraction of *L. monostigma*; (**a**,**b**) *L. monostigma* liver S9 and CTX4A/4B; and (**c**,**d**) *L. monostigma* liver S9 and CTX3C.

**Figure 6:**
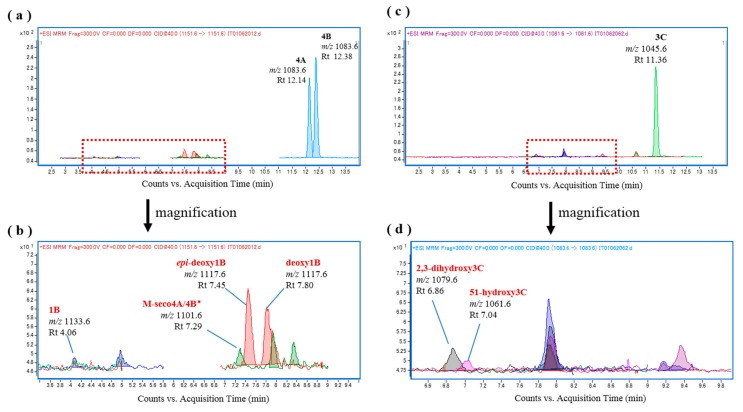
Chromatograms of the in vitro oxidation products of ciguatoxin-4A/4B (CTX4A/4B) and CTX3C following incubation with the liver S9 fraction of *O. punctatus*; (**a**,**b**) *O. punctatus* liver S9 and CTX4A/4B; and (**c**,**d**) *O. punctatus* liver S9 and CTX3C.

Additional representative ciguateric fishes (*L. monostigma* and *O. punctata*) were then used to prepare liver S9 fractions for the in vitro oxidation of CTXs. We found that incubation of CTX4A/4B and CTX3C with the *L. monostigma* S9 fraction produced CTX1B, 52-*epi*-54-deoxyCTX1B, 54-deoxyCTX1B, and 2-hydroxyCTX3C (Figure 5), while incubation of these substrates with the *O. punctata* S9 fraction produced CTX1B, 52-*epi*-54-deoxyCTX1B, 54-deoxyCTX1B, M-*seco*-CTX4A/4B, 2,3-dihydroxyCTX3C, and 51-hydroxyCTX3C (Figure 6). Both the *L. monostigma* and *O. punctatus* S9 fractions were confirmed to be free of these toxins.

**Figure 7:**
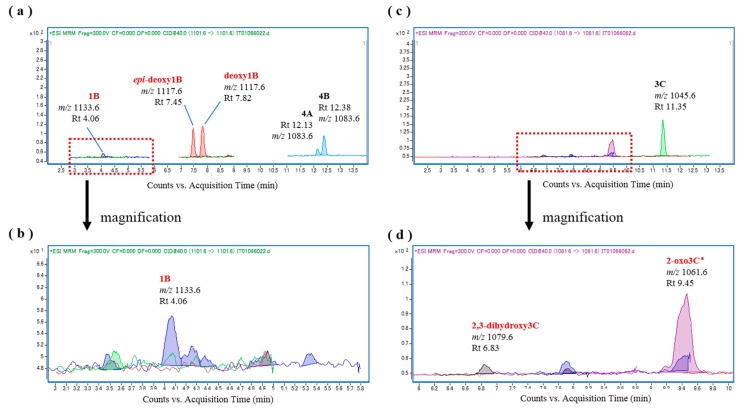
Chromatograms of the in vitro oxidation products of ciguatoxin-4A/4B (CTX4A/4B) and CTX3C following incubation with the liver S9 fraction of *L. gibbus*; (**a**,**b**) *L. gibbus* liver S9 and CTX4A/4B; and (**c**,**d**) *L. gibbus* liver S9 and CTX3C.

**Figure 8:**
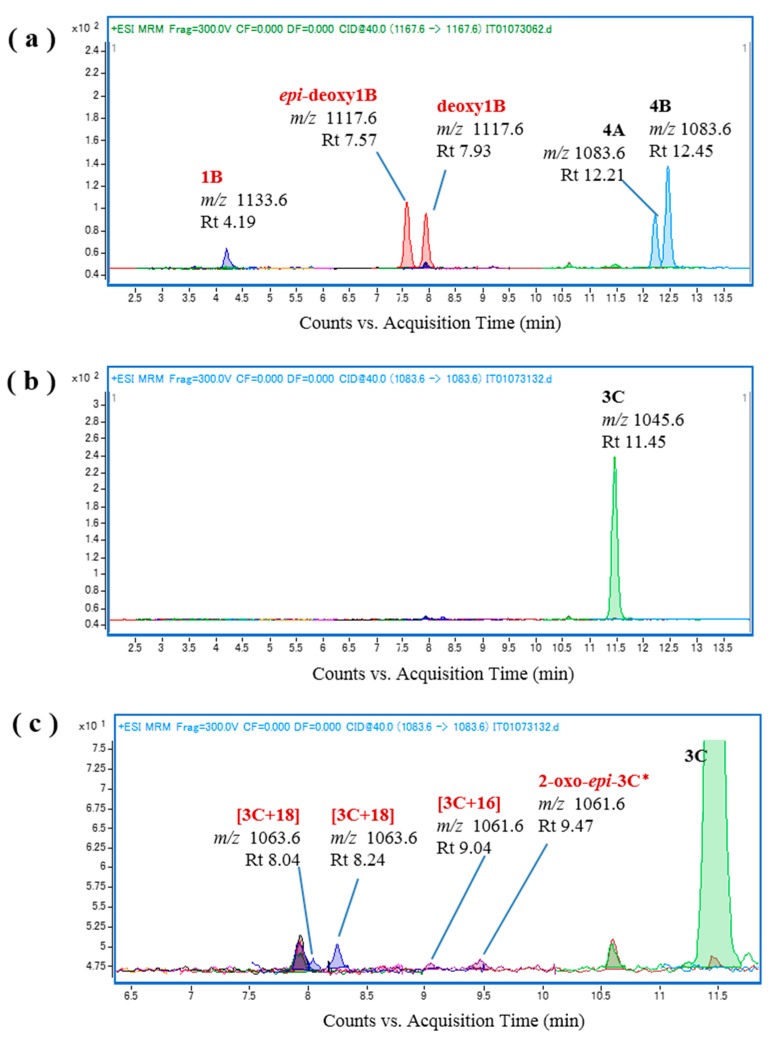
Chromatograms of the in vitro oxidation products of ciguatoxin-4A/4B (CTX4A/4B) and CTX3C following incubation with the liver S9 fraction of *L. fulviflamma*; (**a**) *L. fulviflamma* liver S9 and CTX4A/4B; and (**b**,**c**) *L. fulviflamma* liver S9 and CTX3C.

**Figure 9:**
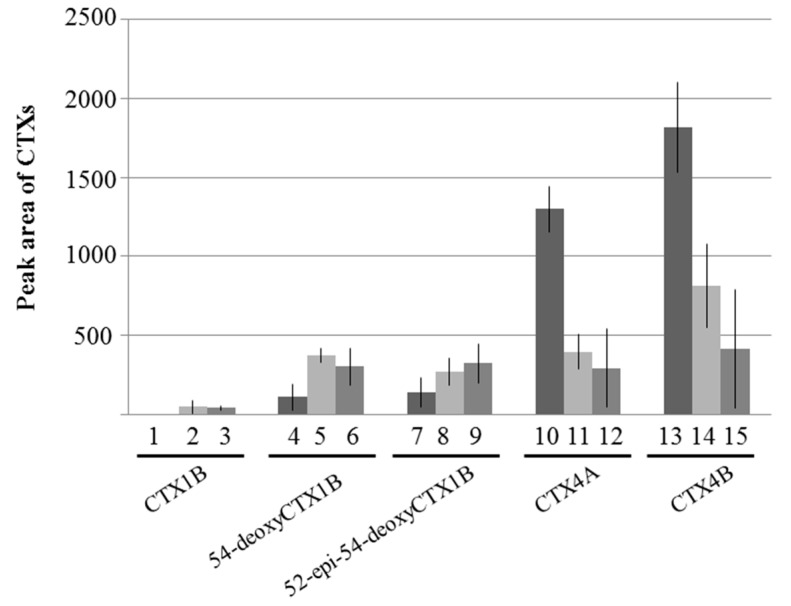
Comparison of the peak areas of various ciguatoxins (CTXs). CTX4A (5 ng) and CTX4B (7.5 ng) were incubated with fish liver S9 at 37 °C for 60 min and the reaction products were identified by liquid chromatography-tandem mass spectrometry (LC-MS/MS) analysis using reference toxins. 1, 4, 7, 10, 13: the in vitro oxidation products following incubation with *L. bohar* liver S9. 2, 5, 8, 11, 14: the in vitro oxidation products following incubation with the liver S9 fraction of *L. gibbus*; and 3, 6, 9, 12, 15: the in vitro oxidation products following incubation with the liver S9 fraction of *L. fulviflamma*. Values represent the means (*n* = 3 specimens/species) ± SD.

Finally, the in vitro CTX oxidation activities of the liver S9 fractions of the non-ciguateric fishes *L. gibbus* and *L. fulviflamma* were evaluated. Interestingly, although the substrates CTX4A and CTX4B were markedly reduced, only very low levels of CTX1B, 54-deoxyCTX1B, and 52-*epi*-54-deoxyCTX1B were produced following incubation with the liver S9 fractions of these species (Figure 7 and Figure 8), none of which were contained in the original fractions. Comparison of the peak areas of the CTXs on the chromatograms for *L. bohar*, *L. gibbus*, and *L. fulviflamma* suggested that CTX4A and CTX4B were converted into some other metabolite that differed from the reference toxins (Figure 9).

## 3. Discussion

**Figure 10:**
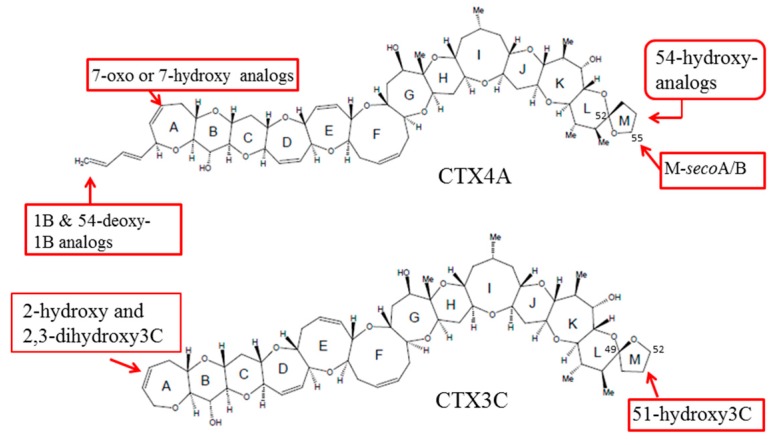
The position and mode of oxidation of ciguatoxin-4A (CTX4A) and CTX3C in recombinant human CYP3A4 (rhCYP3A4) and fish liver S9 fractions.

The bio-oxidation of CTXs has received little attention to date due to the limited availability of purified toxins and the difficulty in developing an appropriate analytical method. In this study, we examined the mechanism that leads to characteristic toxin profiles in ciguateric fishes. We found that both fish liver S9 and human CYP3A4 converted CTX4A and CTX4B to CTX1B, 54-deoxyCTX1B, and 52-*epi*-54-deoxyCTX1B in vitro through oxidation reactions that occurred at the terminal parts of the molecules (Figure 10). This is the first study to provide insight into the biooxidation of CTXs using an enzymatic approach in vitro, providing a basis for further research into the metabolism of CTXs in fish and humans.

Uno et al. [21] previously provided an overview of the CYP superfamily in fish, but the relationship between these enzymes and CTX metabolism remained unclear. In the present study, we evaluated the feasibility of using fish liver S9 and rhCYP3A4 for in vitro oxidation of CTXs, and demonstrated that each of these as well as further fractionated microsomes successfully oxidized these toxins. These findings suggest that a CYP family enzyme in the fish liver is probably involved in the bio-oxidation of CTXs and that CTXs may also be bio-oxidized in the human liver following the consumption of contaminated fish.

CTX1B is the most potent CTX that is currently known with many bioactivities [16] and is the main congener that is found in carnivorous fish. It is believed that this fish metabolite is derived from the oxidation of CTX4A and CTX4B, which are produced by *G. toxicus*, and so the toxicity of CTXs to patients would be increased if this oxidation process occurs in the human liver. In this study, we detected M-*seco*-CTXs and 2-hydroxy analogs as the in vitro oxidation products, the signals for which were assigned based on previous data [10,15]. The toxicity of M-*seco* analogs is unknown but is presumed to be very low because they were isolated from the MBA negative fractions, and so they can be regarded as detoxification products.

In this study, we used four fish species from the genus *Lutjanus* (*L. bohar*, *L. monostigma*, *L. gibbus*, and *L. fulviflamma*). Among these, *L. bohar* is most frequently associated with CFP in Okinawa [21] and other regions [22] of the Pacific, while *L. monostigma* is reputed to be the most toxic fish species in Okinawa. By contrast, no episodes of CFP have been associated with *L. gibbus* or *L. fulviflamma* in Okinawa [21]. Consequently, *L. bohar* and *L. monostigma* are known as ciguateric fishes, while *L. gibbus* and *L. fulviflamma* are known as non-ciguateric fishes in Okinawa. A previous phylogenetic analysis using DNA barcode data of the cytochrome oxidase subunit I and 16S rRNA genes indicated that *L. gibbus* is more closely related to *L. bohar* than to *L. fulviflamma* [23]. However, comparison of the in vitro CTX oxidation activities in these fishes suggested that *L. bohar* and *L. gibbus* have different metabolic pathways for CTX detoxification, which is assumed to begin with an oxidation reaction; and these differences may lead to species-specific toxin profiles in fish. Furthermore, comparison of the liver S9 fractions prepared from five specimens of *L. bohar* demonstrated that there are also individual differences in CTX oxidation activity in this species—though it should be noted that these S9 fractions were prepared from fish obtained from commercial sources and so fluctuations in enzyme activity may have occurred.

Three major ciguatoxins (CTX3C, 51-hydroxyCTX3C, and CTX1B) have been successfully synthesized in previous studies [24,25,26]. However, the preparation of oxidative analogs, such as 54-deoxyCTX1B, 2-hydroxyCTX3C, and 2,3-dihydroxyCTX3C has required their purification from ciguateric fish to date. By contrast, in the present study, CTX1B, 52-*epi*-54-deoxyCTX1B, 54-deoxyCTX1B, 2-hydroxyCTX3C, and 2,3-dihydroxyCTX3C were successfully produced in vitro by enzymatic oxidation, although further improvements may be required for their practical use. Thus, enzymatic preparation of reference toxins to be used in LC-MS analysis from the toxins produced by *G. toxicus* appears to be possible, but only after rigorous testing for optimum conditions and enzymes.

## 4. Materials and Methods

### 4.1. Reagents and Materials

CTX3C and a mixture of CTX4A and CTX4B were prepared from wild and cultured *Gambierdiscus toxicus* dinoflagellates [7,9,11,12]. A standard solution of a CTX mixture containing the toxins shown in Figure S1 was prepared using purified or semi-purified toxins from natural sources and identified by spectral analysis [7,9,10,11,12,13]. The methanol that was used for sample preparation and the LC-MS/MS mobile phase was of LC-MS grade (Kanto Chemical Co., Inc., Tokyo, Japan), while the ammonium formate solution (1 mol/L) and formic acid were of high-performance liquid chromatography (HPLC) grade (Wako Chemical Industry, Ltd., Osaka, Japan). Ultra-pure water was supplied by Milli-Q^®^ Integral Water Purification System for Chemical Analysis (Millipore, Bedford, MA, USA). Supersomes human CYP3A4 + reductase + b5 and the NADPH regenerating system were obtained from Corning (Corning Inc., Corning, NY, USA).

### 4.2. Preparation of Fish Liver S9 and Microsomal Fractions

Eighteen fish specimens from three representative ciguateric fish species (*L. monostigma*, *L. bohar*, and *O. punctatus*) and two non-ciguateric fish species (*L. gibbus* and *L. fulviflamma*) were collected from the Ryukyu Islands in Okinawa Prefecture. The liver was removed from each fish and promptly used for S9 fraction preparation. Individual liver samples were weighed and homogenized in two times the volume of ice-cold buffer (20 mM Tris, 0.15 M KCl, 2 mM ethylenediaminetetraacetic acid (EDTA), 0.5 mM benzamidine, 0.5 mM dithiothreitol (DTT), 0.2 mM phenylmethylsulfonyl fluoride (PMSF), and 10% glycerol) using a glass-Teflon homogenizer. The homogenate was then centrifuged at 9000× *g* for 10 min at 4 °C and the resultant supernatant (S9 fraction) was ultracentrifuged at 100,000× *g* for 60 min at 4 °C. The microsome pellet was resuspended and homogenized in ice-cold buffer. The protein was assayed with a commercially available kit (TaKaRa Bradford Protein Assay Kit; TaKaRa, Japan) according to the manufacturer’s instructions, using bovine serum albumin as a standard. The S9 and microsomal fractions were then stored in small aliquots at −80 °C until required for use. All preparation steps of the enzyme sources were carried out on ice.

### 4.3. In Vitro CTX Oxidation Assay and Sample Preparation for LC-MS/MS Analysis

For the CTX oxidation studies, the incubation mixture (100 μL) included the following compounds: potassium phosphate buffer (0.1 M, pH 7.4); NADPH regeneration system; CTX4A (5 ng) + CTX4B (7.5 ng) or CTX3C (0.5 ng); and fish S9 (0.8 mg protein/mL), fish microsome (0.8 mg protein/mL), or recombinant human CYP3A4 (0.1 mg protein/mL). The reaction mixture was incubated at 37 °C for 60 min and the reaction was then terminated by the addition of 100 μL of acetonitrile. Following this, the mixture was centrifuged, the supernatant was dried, and the residues were re-solubilized in 1 mL of 50% MeOH (*v*/*v*) and applied to an InertSep C18 (100 mg/1 mL) cartridge column (GL Science Inc., Tokyo, Japan) that had previously been treated with 3 mL of methanol and 3 mL of ultra-pure water. The column was washed with 3 mL of 60% methanol (*v*/*v*) and the CTXs were eluted with 2 mL of methanol. The eluates were then dried under a nitrogen stream at 40 °C and dissolved in 1 mL of methanol in preparation for LC-MS/MS analysis.

### 4.4. LC-MS/MS Analysis

The LC-MS/MS system was comprised of an Agilent 1290 HPLC system and an Agilent 6460 Triple Quadrupole MS instrument (Agilent Technologies, Santa Clara, CA, USA). The LC-MS conditions matched those outlined previously [15,16,27] with slight modification.

Each sample solution (5 μL) was injected into and separated with a Zorbax Eclipse Plus C18 column (2.1 × 50 mm, 1.8 μm; Agilent Technologies, Santa Clara, CA, USA) at 40 °C using mobile phases A (5 mM ammonium formate and 0.1% formic acid in water) and B (MeOH). The gradient started at 60% B and was held from 0.00 to 0.25 min; it was then increased to 60–75% B from 0.25 to 0.50 min and 75–90% B from 0.5 to 12.0 min, where it was held until 14.0 min with a flow rate of 0.4 mL/min. The analytical column was washed with methanol for 6 min and then re-equilibrated with 60% B for 3 min with a flow rate of 0.5 mL/min.

The MS parameters were set as follows: electrospray ionization (ESI) positive mode with Agilent Jet Stream; dry gas, 10 L/min of N_2_ at 300 °C; nebulizer gas, N_2_ at 50 psi; sheath gas, 11 L/min of N_2_ at 400 °C; capillary voltage, 5000 V; nozzle voltage, 1000 V; and fragmentor voltage, 300 V. The [M + Na]^+^ ions were used as precursor ions and product ions, with a collision energy of 40 eV, as previously reported [15,16,27].

## Figures and Tables

**Table 1:** The abbreviations and the [M + Na]^+^ (*m*/*z*) value of each CTX congener.

Abbreviations	CTX Congeners	[M + Na]^+^ *m*/*z*	Abbreviations	CTX Congeners	[M + Na]^+^ *m*/*z*
4A	CTX4A	1083.6	3C	CTX3C	1045.6
4B	CTX4B	1083.6	2-oxo3C	2-oxoCTX3C	1061.6
M-*seco*4A/4B	M-*seco*CTX4A/CTX4B	1101.6	2-oxo-*epi*-3C	2-oxo-*epi*-CTX3C	1061.6
deoxy1B	54-deoxyCTX1B	1117.6	2,3-dihydroxy3C	2,3-dihydroxyCTX3C	1079.6
*epi*-deoxy1B	52-*epi*-54-deoxyCTX1B	1117.6	51-hydroxy3C	51-hydroxyCTX3C	1061.6
1B	CTX1B	1133.6	2-hydroxy3C	2-hydroxyCTX3C	1063.6
4-hydroxy-7-oxo-1B	4-hydroxy-7-oxo-CTX1B	1167.6			

## Supplementary Materials

The following are available online at www.mdpi.com/2072-6651/9/7/205/s1. Figure S1: Chromatograms, retention times (Rts), and *m*/*z* values for the ciguatoxins (CTXs) that were used as substrates for in vitro oxidation; Figure S2: Chromatograms of the in vitro oxidation products of ciguatoxin-4A/4B (CTX4A/4B) following incubation with recombinant human CYP3A4 (rhCYP3A4); Figure S3: Chromatograms of the in vitro oxidation products of ciguatoxin-4A/4B (CTX4A/4B) following incubation with human liver microsomes; Figure S4: Chromatograms of the in vitro oxidation products of ciguatoxin-4A/4B (CTX4A/4B) following incubation with the S9 fractions from four different *Lutjanus bohar* specimens; Figure S5: Chromatograms of the S9 fractions of four different *Lutjanus bohar* specimens; Table S1: Body size of fish samples used in this study.
