# Supplementary material for: Biooxidation of Ciguatoxins Leads to Species-Specific Toxin Profiles
Source: Toxins (Basel). 2017 Jun 29;9(7):205. doi: 10.3390/toxins9070205 (PMC5535152; doi:10.3390/toxins9070205)
Supplement: Supplementary file 1 [file toxins-09-00205-s001.pdf]

Supplementary Materials: Biooxidation of Ciguatoxins Leads to Species-Specific Toxin Profiles

Tsuyoshi Ikehara, Kyoko Kuniyoshi, Naomasa Oshiro and Takeshi Yasumoto

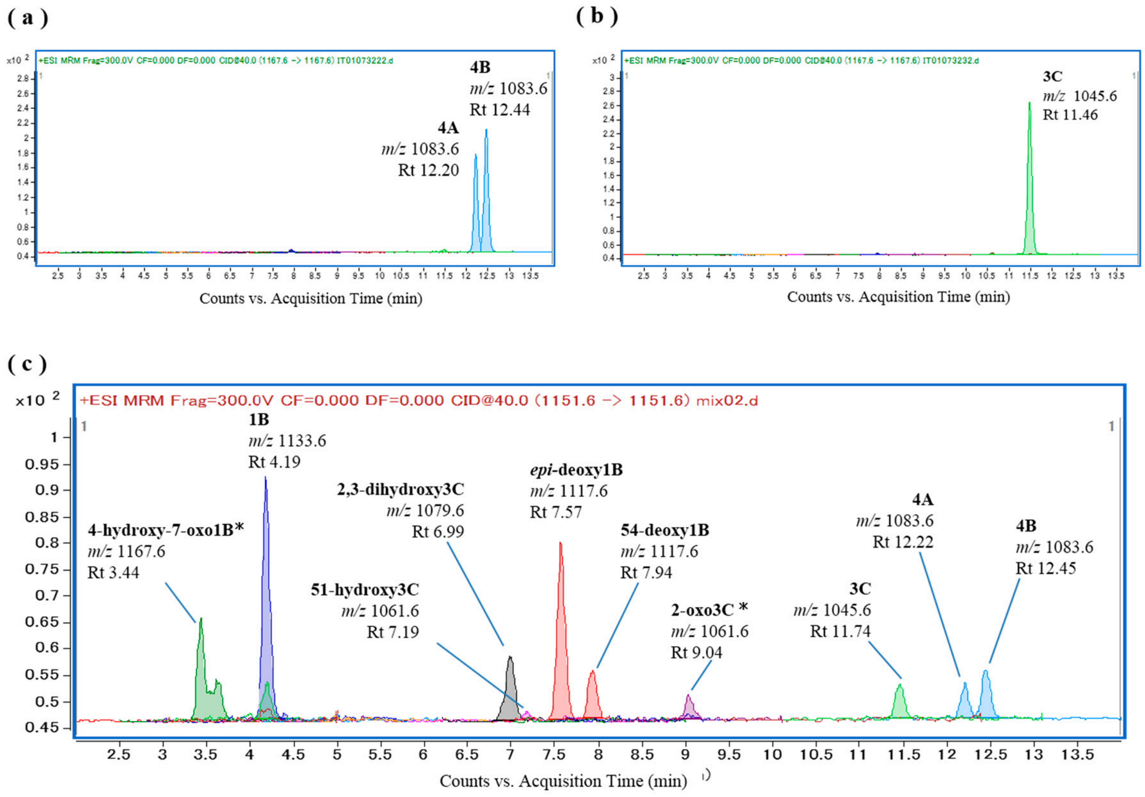

Figure S1. Chromatograms, retention times (Rts), and *m*/*z* values for the ciguatoxins (CTXs) that were used as substrates for *in vitro* oxidation. (**a**) CTX4A/4B; (**b**) CTX3C; and (**c**) reference toxins for liquid chromatography-tandem mass spectrometry (LC-MS/MS) analysis. 4A: CTX4A; 4B: CTX4B; 3C: CTX3C; 4-hydroxy-7-oxo-1B: 4-hydroxy-7-oxo-CTX1B; 1B: CTX1B; 2,3-dihydroxy3C: 2,3-dihydroxyCTX3C; 51-hydroxy3C: 51-hydroxyCTX3C; *epi*-deoxy1B: 52-epi-54-deoxyCTX1B; deoxy1B: 54-deoxyCTX1B; 2-oxo3C: 2-oxo-CTX3C.

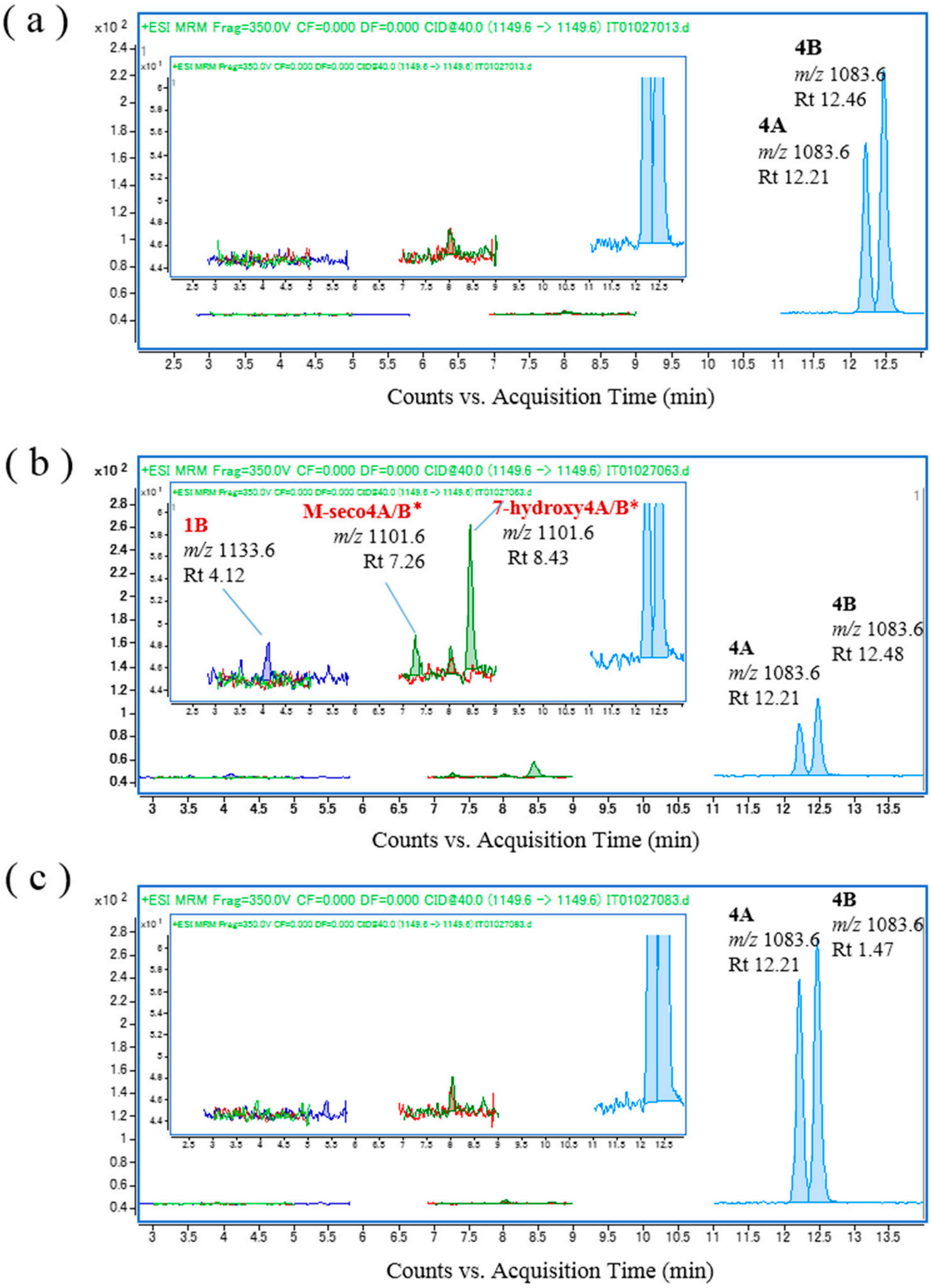

Figure S2. Chromatograms of the *in vitro* oxidation products of ciguatoxin-4A/4B (CTX4A/4B) following incubation with recombinant human CYP3A4 (rhCYP3A4). Products are shown (**a**) at 0 min, (**b**) at 60 min, and (**c**) at 60 min without rhCYP3A4. The inset in each panel shows a magnified view of the chromatogram. 4A: CTX4A; 4B: CTX4B; 1B: CTX1B; M-*seco*4A/4B: M-*seco*CTX4A/4B; 7-hydroxy4A/4B: 7-hydroxyCTX4A/4B.

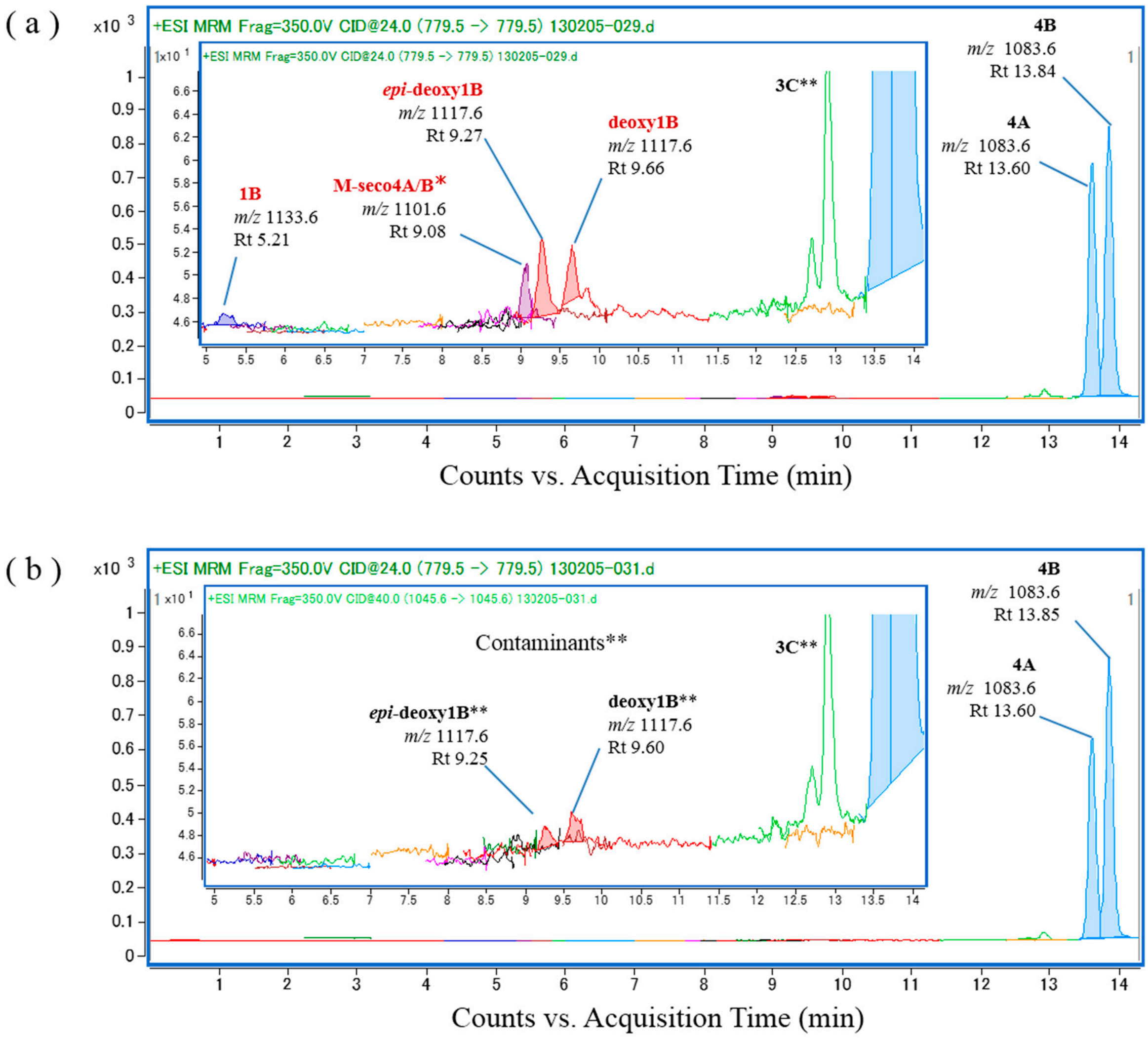

Figure S3. Chromatograms of the *in vitro* oxidation products of ciguatoxin-4A/4B (CTX4A/4B) following incubation with human liver microsomes. (**a**) Untreated microsomes and (**b**) heat-treated microsomes at 90 °C for 5 min. The inset in each panel shows a magnified view of the chromatogram. ** The 4A/4B substrate contained trace amounts of CTX3C, *epi*-deoxy1B, and deoxy1B (*m/z* 1117). 1B: CTX1B; M-*seco*4A/4B: M-*seco*CTX4A/4B; *epi*-deoxy1B: 52-epi-54-deoxyCTX1B; deoxy1B: 54-deoxyCTX1B; 3C: CTX3C; 4A: CTX4A; 4B: CTX4B.

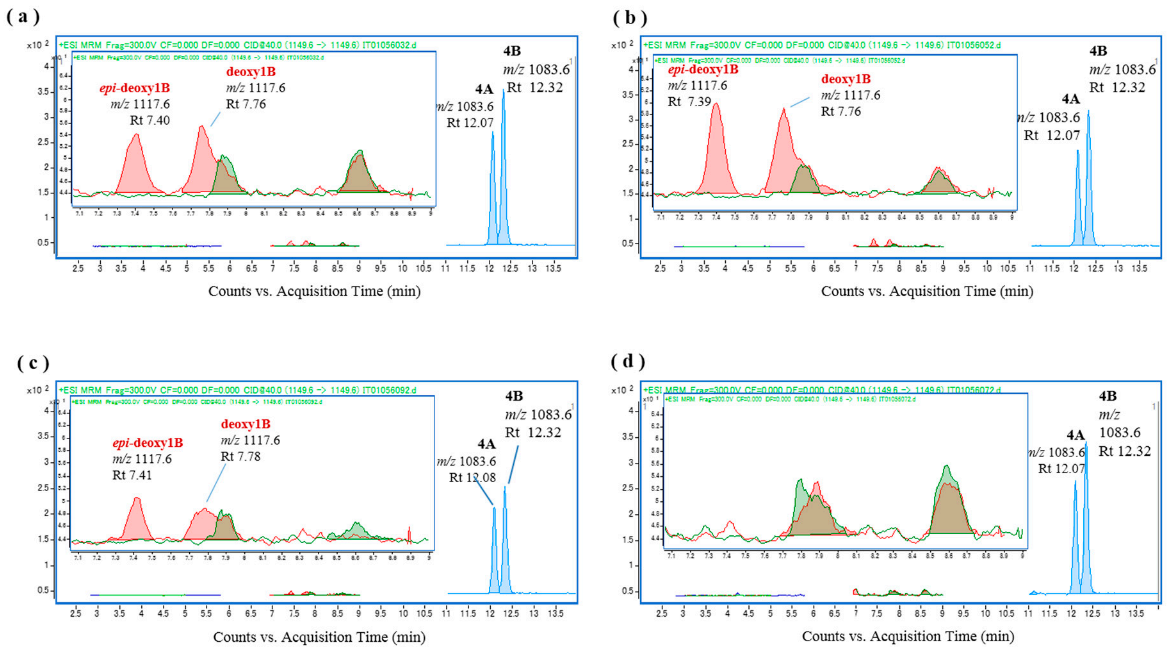

Figure S4. Chromatograms of the *in vitro* oxidation products of ciguatoxin-4A/4B (CTX4A/4B) following incubation with the S9 fractions from four different *Lutjanus bohar* specimens. (**a**) Specimen of 1.5 kg body weight (BW) and 45 cm total length (TL); (**b**) specimen of 1.1 kg BW and 42 cm TL; (**c**) specimen of 3.8 kg BW and 62 cm TL; and (**d**) specimen of 0.5 kg BW and 41 cm TL. The inset in each panel shows a magnified view of the chromatogram. *epi*-deoxy1B: 52-epi-54-deoxyCTX1B; deoxy1B: 54-deoxyCTX1B; 4A: CTX4A; 4B: CTX4B.

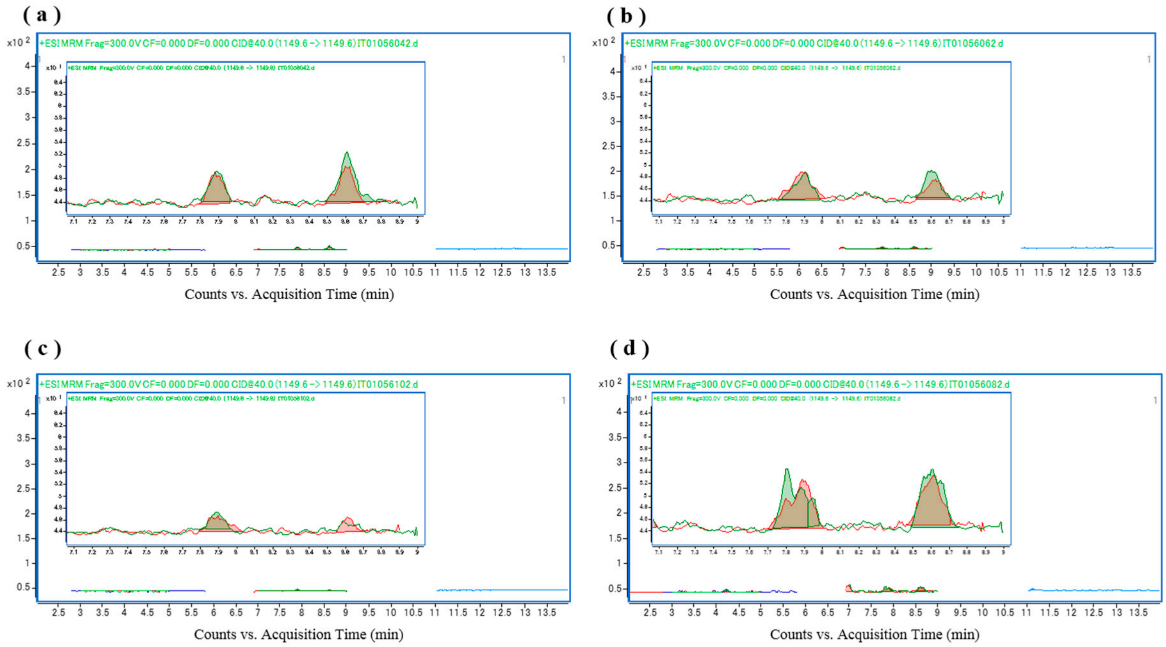

Figure S5. Chromatograms of the S9 fractions of four different *Lutjanus bohar* specimens. (**a**) Specimen of 1.5 kg body weight (BW) and 45 cm total length (TL); (**b**) specimen of 1.1 kg BW and 42 cm TL; (**c**) specimen of 3.8 kg BW and 62 cm TL; and (**d**) specimen of 0.5 kg BW and 41 cm TL. The inset in each panel shows a magnified view of the chromatogram.

Table S1. Body size of fish samples used in this study

Sample No.	Species	Sample size	
		**Body weight (kg)**	**Body length (cm)**
1	*Lutjanus bohar*	3.80	63.0
2		1.50	45.0
3		1.10	42.0
4		3.80	62.0
5		0.50	31.0
6	*Lutjanus monostigma*	0.50	30.0
7		1.70	47.0
8	*Oplegnathus punctatus*	1.10	30.0
9		2.00	50.0
10	*Lutjanus gibbus*	0.88	37.0
11		0.82	38.0
12		0.72	36.0
13		0.86	38.5
14		0.84	38.0
15	*Lutjanus fulviflamma*	0.42	29.0
16		0.36	27.5
17		0.37	26.0
18		0.42	28.0
